# Malvidin-3-O-Glucoside Mitigates α-Syn and MPTP Co-Induced Oxidative Stress and Apoptosis in Human Microglial HMC3 Cells

**DOI:** 10.3390/ijms252312733

**Published:** 2024-11-27

**Authors:** Rachit Sood, Sanjay, Sung-Ung Kang, Na Young Yoon, Hae-Jeung Lee

**Affiliations:** 1 Department of Food and Nutrition, College of BioNano Technology, Gachon University, Seongnam 13120, Republic of Korea; rachitsood1998@gmail.com (R.S.); sanjay.monga4@gmail.com (S.); 2 Institute for Aging and Clinical Nutrition Research, Gachon University, Seongnam 13120, Republic of Korea; 3 Neuroregeneration and Stem Cell Programs, Institute for Cell Engineering, Johns Hopkins University School of Medicine, Baltimore, MD 21205, USA; skang34@jhmi.edu; 4 Department of Neurology, Johns Hopkins University School of Medicine, Baltimore, MD 21205, USA; 5 Food Safety and Processing Research Division, National Institute of Fisheries Science, Busan 46083, Republic of Korea; dbssud@korea.kr; 6 Department of Health Sciences and Technology, Gachon Advanced Institute for Health Science and Technology (GAIHST), Gachon University, Incheon 21999, Republic of Korea

**Keywords:** Parkinson’s disease, anthocyanin, malvidin-3-O-glucoside, anti-apoptotic, antioxidation, anti-inflammation, Nrf2/HO-1 signaling, neurodegenerative diseases

## Abstract

Parkinson’s disease (PD) is a widespread age-related neurodegenerative disorder characterized by the presence of an aggregated protein, α-synuclein (α-syn), which is encoded by the *SNCA* gene and localized to presynaptic terminals in a normal human brain. The α-syn aggregation is induced by the 1-methyl-4-phenyl-1,2,3,6-tetrahydropyridine (MPTP) mitochondrial neurotoxin and is therefore used to mimic PD-like pathology in various in vitro and in vivo models. However, in vitro PD-like pathology using α-syn and MPTP in human microglial cells has not yet been reported. Malvidin-3-O-glucoside (M3G) is a major anthocyanin primarily responsible for pigmentation in various fruits and beverages and has been reported to possess various bioactivities. However, the neuroprotective effects of M3G in humanized in vitro PD-like pathologies have not been reported. Therefore, individual and co-treatments of α-syn and MPTP in a human microglial (HMC3) cell line were used to establish a humanized PD-like pathology model in vitro. The individual treatments were significantly less cytotoxic when compared to the α-syn and MPTP co-treatment. This study examined the neuroprotective effects of M3G by treating HMC3 cells with α-syn (8 μg/mL) and MPTP (2 mM) individually or in a co-treatment in the presence or absence of M3G (50 μM). M3G demonstrated anti-apoptotic, anti-inflammatory, and antioxidative properties against the α-syn- and MPTP-generated humanized in vitro PD-like pathology. This study determined that the cytoprotective effects of M3G are mediated by nuclear factor erythroid 2-related factor 2 (Nrf2)/heme oxygenase (HO)-1 signaling.

## 1. Introduction

Parkinson’s disease (PD) is the most common age-related neurodegenerative disorder (ND) after Alzheimer’s disease (AD) and currently affects more than ten million individuals globally [1]. Pathologically, PD is characterized by the accumulation of neuropathological hallmarks such as Lewy bodies and Lewy neurites in dopamine-producing (dopaminergic) neurons, which results in the loss of these neurons in the substantia nigra pars compacta (SNpc) region of the midbrain [2]. Clinically, PD is characterized by common motor and non-motor impairments, such as bradykinesia, stooping posture, anxiety, resting tremor, rigidity, cognitive impairments, autonomic dysfunction, depression, and sleep disorders [3].

α-synuclein (α-syn), an intracellular protein primarily composed of 140 amino acids, is the major hallmark of PD and is localized to the presynaptic terminals (axons and synapses) of a normal human brain [4]. These proteins are deposited as fibrils and are the primary protein detected in Lewy bodies and Lewy neurites [5]. Because of the unfolded native structure of α-syn, it is highly prone to self-aggregation, resulting in the formation of oligomers [6]. Additionally, several mutations in *SNCA*, such as p.A53T and p.A30P, are more susceptible to the formation of α-syn oligomer protofibrillar intermediates [7]. Although α-syn fibrils are known to contribute to PD pathology, the exact reason for their aggregation and fibril formation remains unknown. Moreover, the signaling cascades activated by this aggregation, which eventually result in cell injury and death, are unknown.

Although numerous transgenic PD models overexpressing mutated and wild-type α-syn have been reported, none of them have been able to completely mimic the key pathological and neurotoxic characteristics of PD [8]. 1-methyl-4-phenyl-1,2,3,6-tetrahydropyridine (MPTP; a mitochondrial neurotoxin) is reported to induce α-syn aggregation in BV2 microglial cells [9] and several mouse models [10,11], thus amplifying α-syn-mediated neurodegeneration [12] and inhibiting mitochondrial complex I [13]. Mitochondrial dysfunction, as a result of MPTP, has been reported to affect α-syn and vice versa, thus worsening PD symptoms [14]. Considering these factors, the MPTP mouse model is commonly used to replicate the neurotoxicity and pathophysiology of PD in vivo. Hence, this study aims to determine the same effects in an in vitro PD-like pathology model.

Programmed cell death, also known as apoptosis, plays a vital role in the pathogenesis of PD. The process of apoptosis is aggravated by various factors in PD, including α-syn aggregation and exposure to several toxins, such as MPTP, rotenone, and 6-OHDA [15]. α-syn aggregation disrupts cellular homeostasis, which leads to mitochondrial dysfunction and ultimately oxidative stress, both of which trigger different apoptotic pathways. Similarly, MPTP, when metabolized to its toxic form MPP^+^, induces microglia-mediated apoptotic death in PD by triggering oxidative stress and neuroinflammation, eventually contributing to dopaminergic neuron degeneration [16]. Microglial apoptosis negatively affects dopaminergic neurons by disrupting the neuronal network, reducing neuroprotection, altering toxic aggregate clearance, impairing inflammatory responses, and ultimately enhancing the susceptibility to neurodegeneration [17]. Together, these processes contribute to progressive PD pathophysiology, underlining apoptosis as a primary mechanism in PD pathology. Thus, a thorough understanding of these mechanisms is critical for the development of therapeutic strategies aimed at modulating apoptosis and preventing cell death in patients with PD.

Recently, the roles of various neuroinflammatory processes in nigrostriatal dopaminergic neuronal cell loss in PD have received attention. Microglia, major brain resident macrophages, play a vital role in inflammation-related processes and have been found to be densely populated in the SNpc region of the brain. Upon activation, these microglia transform from resting striated bodies into large amoeboid and round bodies [18]. Contrary to the beneficial housekeeping role of resting and slightly activated microglia, activated microglia located in the SNpc are involved in PD pathogenesis [17] and are a chronic source of various pro-inflammatory cytokines such as interleukin (IL)-1β, tumor necrosis factor (TNF)-α, IL-6, and reactive oxygen species (ROS) [19,20], which induce progressive neuronal damage. Microglia are also associated with the chronic nature of ND diseases, particularly PD. Although the mechanisms driving these phenomena are being actively studied, the microglial response to neuronal damage (reactive microgliosis) and ROS have been suggested as the primary mechanisms of chronic and neurotoxic microglial activation, specifically in PD.

Recent studies have reported that oxidative stress resulting from mitochondrial impairment is a major factor contributing to the onset of PD [21]. Exposure to ROS activates antioxidants with the help of nuclear factor erythroid 2-related factor 2 (Nrf2; transcription factor), which is the prime regulator of the cellular redox response [22]. Nrf2 regulates various downstream targets, such as heme oxygenase (HO), catalase (CAT), superoxide dismutase (SOD), and glutathione peroxidase (GPx), which are important antioxidants in nearly all cells [23]. The overexpression of these antioxidants prevents lipid peroxidation and H_2_O_2_ accumulation and ultimately prevents neuronal loss during neurotoxic conditions [24].

Malvidin-3-O-glucoside (M3G), a glycoside of the aglycon malvidin, comprises > 75% of the total polyphenolic content in red grapes (*Vitis vinifera*), red wine, and other colored fruit skins [25]. Additionally, various other derivatives of malvidin are abundantly found in functional foods, such as bilberries, blueberries, and raspberries. Although M3G is primarily responsible for providing color and flavor in most foods, fruits, and beverages, it is much more than just a mere coloring agent; it has been reported to display excellent bioactivities in both in vivo and in vitro studies, including anti-cancer [26], anti-diabetic [27], anti-inflammatory [28], antioxidative [29], and anti-amyloidogenic [30] properties. Several studies have reported the neuroprotective effects of M3G in different cell lines; however, its neuroprotective effects on the α-syn- and MPTP-induced human microglia (HMC3) cells are yet to be explored. MPTP is an effective inducer in HMC3 (microglial) cells because it mimics the key pathological hallmarks of PD by inducing neuroinflammation, activating microglia, and releasing pro-inflammatory cytokines and ROS [31,32]. This reflects the chronic inflammation observed in PD, thus helping to examine the role of neuroinflammation and toxic feedback loops in PD progression. Likewise, using α-syn as an inducer in HMC3 cells is valuable because it activates microglia, thereby triggering a pro-inflammatory response and generating a feedback loop of inflammation and neurotoxicity [17,33], which helps in studying the key signaling pathways involved in neuroinflammation, offering insights for potential therapeutic targets. Considering these factors, this study aims to establish a humanized in vitro PD-like pathology model using α-syn and MPTP co-treated HMC3 cells and examine the cytoprotective effects of M3G by assessing various PD-related factors such as apoptosis, inflammation, and oxidative stress.

## 2. Results

### 2.1. α-Syn- and MPTP-Induced Cytotoxicity in HMC3 Cells

**Figure 1:**
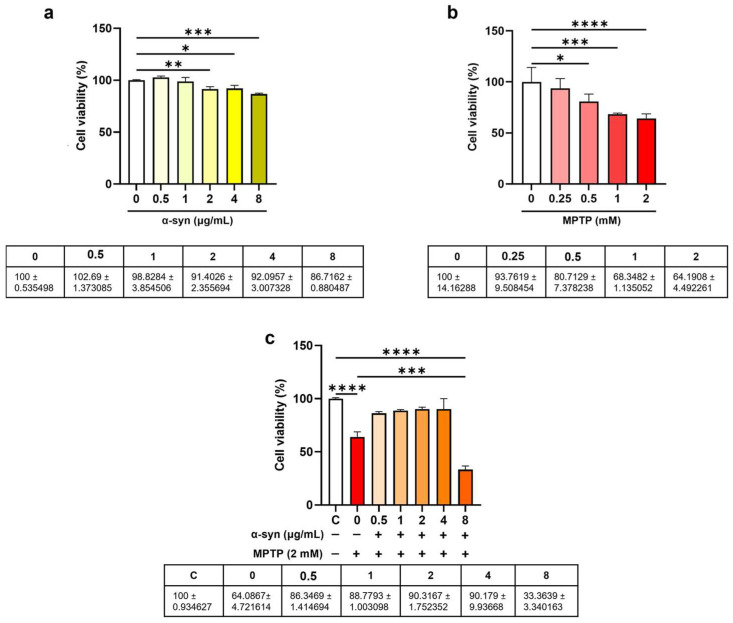
α-syn- and MPTP-induced cytotoxicity in HMC3 cells. HMC3 cells treated with (**a**) different concentrations of α-syn (0.5, 1, 2, 4, and 8 μg/mL), (**b**) different concentrations of MPTP (0.25, 0.5, 1, and 2 mM), and (**c**) individual or co-treatment of MPTP (2 mM) in the presence of various concentrations of α-syn (0.5, 1, 2, 4, and 8 μg/mL) for 24 h. C, control; α-syn, α-synuclein; MPTP, 1-methyl-4-phenyl-1,2,3,6-tetrahydropyridine. Experiments were conducted in triplicate, and all the data are shown as the mean ± standard deviation (SD). * *p* < 0.05, ** *p* < 0.01, *** *p* < 0.001, and **** *p* < 0.0001.

As per the CCK-8 assay, α-syn was significantly cytotoxic at concentrations of 2, 4, and 8 μg/mL (*p <* 0.05), with 8 μg/mL demonstrating the most cytotoxicity (Figure 1a). Similarly, MPTP-induced cytotoxicity in HMC3 cells occurred in a concentration-dependent manner, with 2 mM being the optimal concentration (Figure 1b).

As shown in Figure 1c, 8 μg/mL of α-syn and 2 mM of MPTP were the optimal concentrations for cytotoxicity induction, and these concentrations were used for the remaining experiments. Moreover, in comparison to the individual treatment group, the co-treatment of MPTP (2 mM) with various concentrations of α-syn (0.5, 1, 2, 4, and 8 μg/mL) demonstrated a greater statistical significance, indicating that the combined treatment presents greater cytotoxicity. The combined treatment group displayed a more pronounced reduction in cell viability.

### 2.2. M3G Ameliorates α-Syn- and MPTP-Induced Cell Death (Apoptosis) in HMC3 Cells

**Figure 2:**
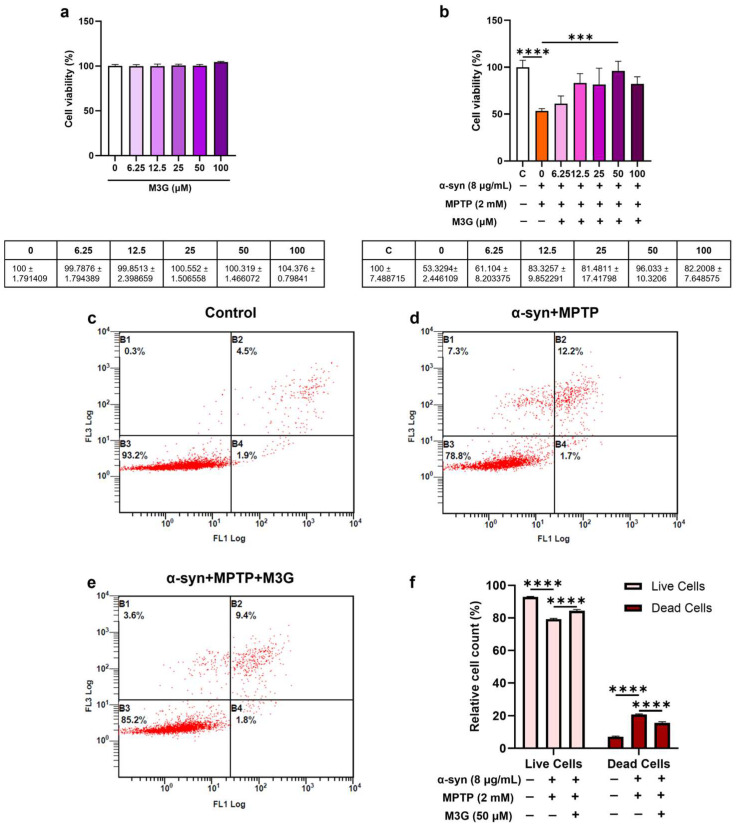
M3G protects HMC3 cells from α-syn- and MPTP-induced cytotoxicity. HMC3 cells (**a**) treated with different concentrations of M3G (6.25, 12.5, 25, 50, and 100 μM) and (**b**) co-treated with α-syn (8 μg/mL) and MPTP (2 mM) in the presence or absence of various concentrations of M3G (6.25, 12.5, 25, 50, and 100 μM). (**c**–**f**) HMC3 cells co-treated with α-syn (8 μg/mL) and MPTP (2 mM) in the presence or absence of M3G (50 μM). C, control; α-syn, α-synuclein; MPTP, 1-methyl-4-phenyl-1,2,3,6-tetrahydropyridine; M3G, malvidin-3-O-glucoside. Experiments were conducted in triplicate, and all the data are shown as the mean ± standard deviation (SD). *** *p* < 0.001 and **** *p* < 0.0001.

HMC3 cells were treated with various concentrations of M3G. The results revealed that M3G was non-toxic to HMC3 cells at all tested concentrations (6.25, 12.5, 25, 50, and 100 μM; Figure 2a). The cytoprotective potential of M3G against the α-syn- and MPTP-generated in vitro humanized PD-like pathology was examined. The results showed that M3G significantly reduced α-syn- and MPTP-induced cytotoxicity in a concentration-dependent manner in the co-treatment group. It was determined that 50 μM of M3G was the optimal concentration, and this was used for further experiments (Figure 2b).

To verify the CCK-8 assay results and determine whether α-syn- and MPTP-induced cell death is a result of apoptosis, the above experiments were repeated using an annexin V apoptosis assay kit. The relative cell count of the live and apoptotic cells was observed and calculated. The relative cell counts of viable cells decreased, whereas the relative cell counts of dead cells significantly increased in the α-syn and MPTP co-treatment group. These cytotoxic effects were ameliorated in the presence of M3G, where the relative cell count of viable cells increased significantly and dead cells decreased when compared to the α-syn and MPTP co-treatment group, thus indicating the anti-apoptotic and cytoprotective properties of M3G (Figure 2c–f).

**Figure 3:**
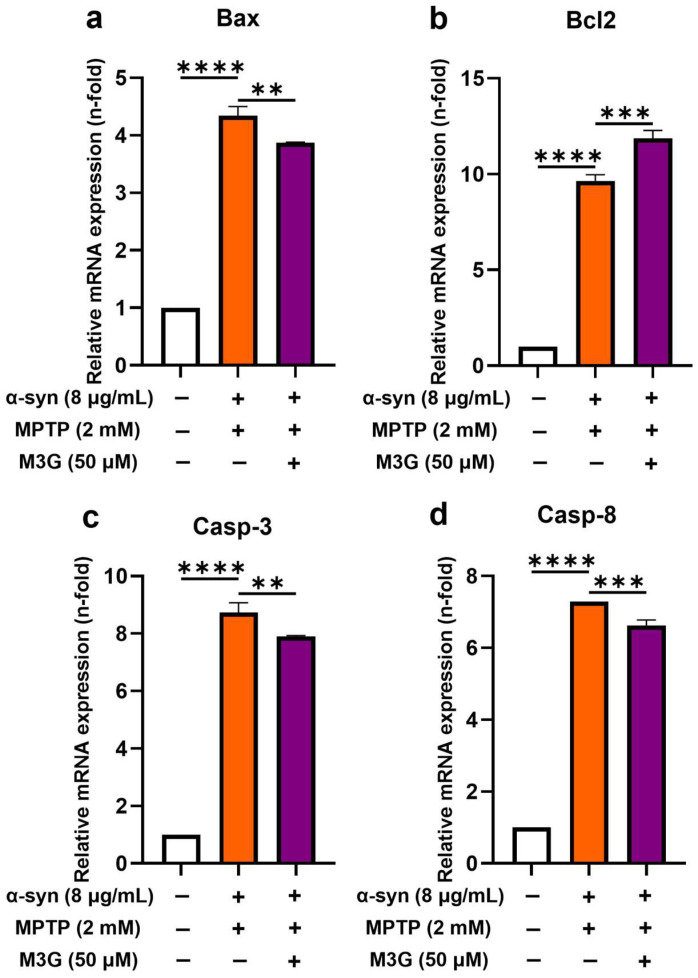
M3G ameliorates α-syn- and MPTP-induced apoptosis by upregulating the transcriptional expression level of the anti-apoptotic marker and downregulating the pro-apoptotic markers in HMC3 cells. HMC3 cells were co-treated with α-syn (8 μg/mL) and MPTP (2 mM) in the presence or absence of M3G (50 μM), and the mRNA expression levels of (**a**) Bax, (**b**) Bcl2, (**c**) Casp-3, and (**d**) Casp-8 were analyzed by RT-PCR. α-syn, α-synuclein; MPTP, 1-methyl-4-phenyl-1,2,3,6-tetrahydropyridine; M3G, malvidin-3-O-glucoside; Bax, Bcl-2-Associated X-protein; Bcl2, B-cell lymphoma 2; Casp, caspase. Experiments were conducted in triplicate, and all the data are shown as the mean ± standard deviation (SD). ** *p* < 0.01, *** *p* < 0.001, and **** *p* < 0.0001.

**Figure 4:**
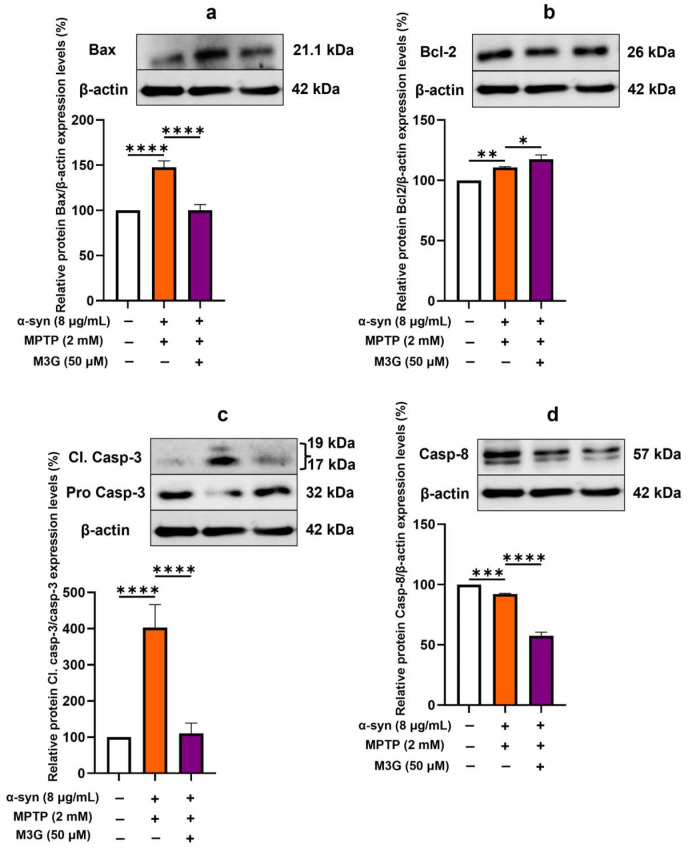
M3G ameliorates α-syn- and MPTP-induced apoptosis by upregulating the translational expression level of the anti-apoptotic marker and downregulating the pro-apoptotic markers in HMC3 cells. HMC3 cells were co-treated with α-syn (8 μg/mL) and MPTP (2 mM) in the presence or absence of M3G (50 μM). The protein expression level of (**a**) Bax, (**b**) Bcl2, (**c**) cleaved casp-3/pro-casp-3, and (**d**) casp-8 were analyzed by Western blot. α-syn, α-synuclein; MPTP, 1-methyl-4-phenyl-1,2,3,6-tetrahydropyridine; M3G, malvidin-3-O-glucoside; Bax, Bcl-2-Associated X-protein; Bcl2, B-cell lymphoma 2; Casp, caspase. Experiments were conducted in triplicate, and all the data are shown as the mean ± standard deviation (SD). * *p* < 0.05, ** *p* < 0.01, *** *p* < 0.001, and **** *p* < 0.0001.

Although microglial activation and the occurrence of DAMs are key contributors to PD pathology, evidence for microglial cell death in PD brains is limited [34]. Some studies have suggested that prolonged activation can lead to oxidative stress and apoptosis, thereby altering the inflammatory environment and contributing to neurodegeneration [35]. Nonetheless, since microglial activation produces pro-inflammatory cytokines and ROS, leading to neuronal damage, it is important to consider apoptosis when analyzing factors related to MPTP- and α-syn-induced PD pathology. Considering these factors, the mRNA and protein expression levels of different pro-apoptotic markers (Bax, casp-3, and casp-8) and the anti-apoptotic marker Bcl-2 were also studied to understand the mechanism behind α-syn- and MPTP-induced apoptotic cell death. The mRNA expression levels of the pro-apoptotic markers were found to be upregulated in the α-syn and MPTP co-treatment group. These were then significantly downregulated in the presence of M3G (*p <* 0.05; Figure 3a,c,d). The anti-apoptotic marker Bcl-2 was significantly upregulated in the M3G-only group compared to in the α-syn and MPTP co-treatment groups (Figure 3b). Furthermore, the protein expression levels of the pro-apoptotic markers, such as Bax, casp-8, and cleaved casp-3/pro-casp-3, were significantly downregulated, and the anti-apoptotic marker, Bax, was significantly upregulated in the M3G-only treatment group when compared to the α-syn and MPTP co-treatment group. However, the elevated protein expression levels of casp-8 in the α-syn and MPTP co-treatment group could not be observed; however, the M3G-only treatment group significantly reduced its protein expression level when compared to the α-syn and MPTP co-treatment group (Figure 4a–d). These results suggest that M3G displays anti-apoptotic properties in α-syn- and MPTP-induced HMC3 cells.

### 2.3. M3G Downregulates α-Syn- and MPTP-Induced Pro-Inflammatory Cytokines and Upregulates Anti-Inflammatory Cytokine Expression

**Figure 5:**
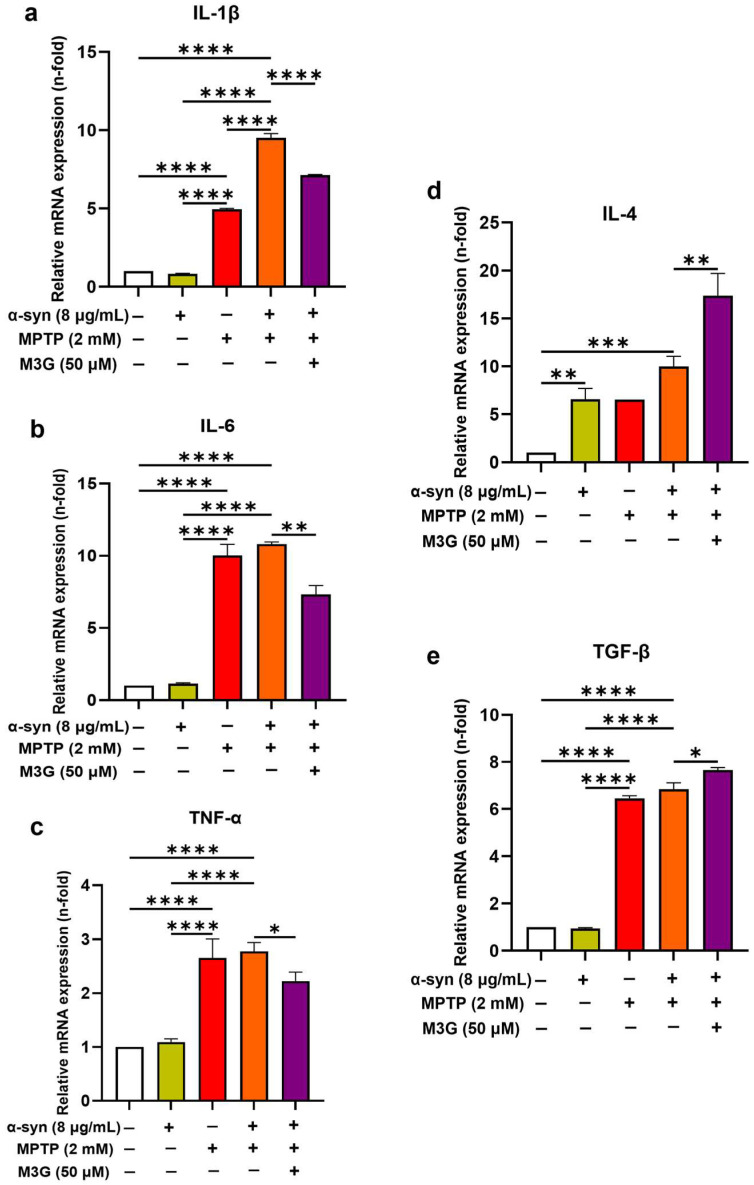
M3G downregulates α-syn- and MPTP-induced pro-inflammatory cytokines and upregulates the anti-inflammatory cytokine expressions in HMC3 cells. HMC3 cells were treated with α-syn (8 μg/mL) and MPTP (2 mM) individually or co-treated with α-syn (8 μg/mL) and MPTP (2 mM) in the presence or absence of M3G (50 μM) for 24 h, and the mRNA expression of the pro-inflammatory cytokines (**a**) IL-1β, (**b**) IL-6, and (**c**) TNF-α and the anti-inflammatory cytokines (**d**) IL-4 and (**e**) TGF-β was analyzed by RT-PCR. α-syn, α-synuclein; MPTP, 1-methyl-4-phenyl-1,2,3,6-tetrahydropyridine; M3G, malvidin-3-O-glucoside; IL, interleukin; TNF-α, tumor necrosis factor-alpha; TGF-β, transforming growth factor-beta. Experiments were conducted in triplicate, and all the data are shown as the mean ± standard deviation (SD). * *p* < 0.05, ** *p* < 0.01, *** *p* < 0.001, and **** *p* < 0.0001.

Inflammation is the primary response to any sort of cellular stress. Therefore, after analyzing the cytoprotective properties of M3G, the anti-inflammatory effects of M3G against the α-syn- and MPTP-induced inflammatory response were assessed. The mRNA expression levels of pro-inflammatory cytokines such as IL-1β, IL-6, and TNF-α were unaffected in the α-syn individual treatment group but showed significant upregulation in the MPTP individual treatment group and displayed even higher upregulation in the α-syn and MPTP co-treatment group. However, the presence of M3G downregulated the α-syn- and MPTP-induced mRNA expression levels of all these pro-inflammatory cytokines significantly (*p* < 0.05; Figure 5a–c). To determine if the M3G individual treatment affects inflammation in HMC3 cells, the cells were treated with M3G alone (50 µM). The results demonstrated that M3G alone does not induce the expression of pro-inflammatory cytokines (IL-1β, IL-6, and TNF-α) but significantly upregulates the expression of anti-inflammatory cytokines (IL-4 and TGF-β) (*p* < 0.05; Supplementary Figure S1).

Additionally, the mRNA expression levels of anti-inflammatory cytokines such as IL-4 and transforming growth factor (TGF)-β showed promising results, as M3G was found to upregulate their mRNA expression levels compared to the α-syn and MPTP co-treatment group (Figure 5d,e). These findings suggest the potential anti-inflammatory properties of M3G in ameliorating the α-syn- and MPTP-induced inflammatory response and promoting anti-inflammatory cytokine expression.

### 2.4. M3G Attenuates α-Syn- and MPTP-Induced ROS Production

**Figure 6:**
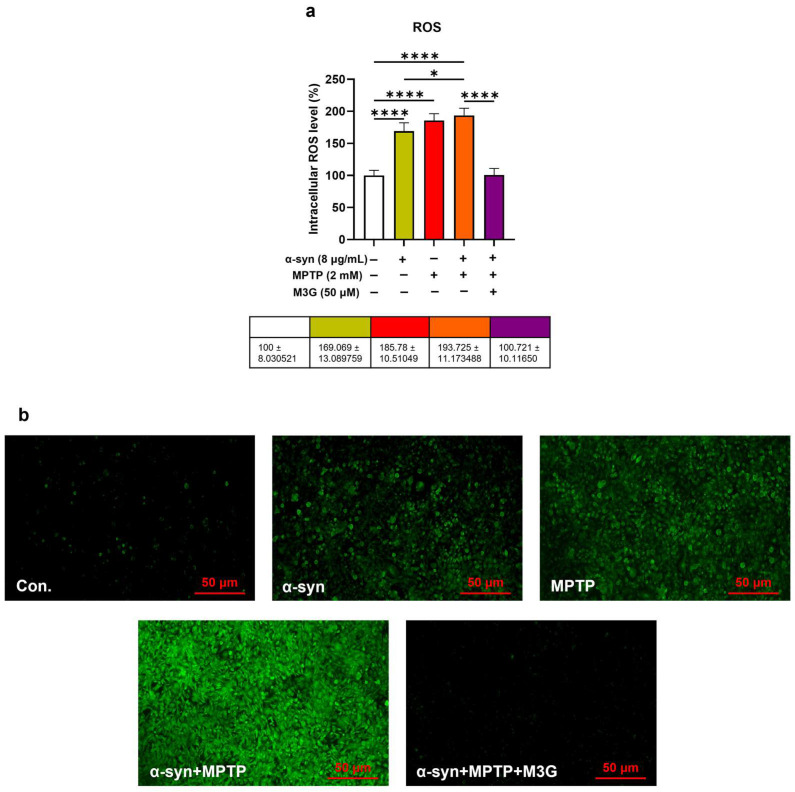
M3G attenuates α-syn- and MPTP-induced reactive oxygen species (ROS) production. HMC3 cells were individually or co-treated with α-syn (8 μg/mL) and MPTP (2 mM) in the presence or absence of M3G (50 μM). Intracellular ROS levels were determined using the DCFH-DA assay through (**a**) a microplate reader and (**b**) fluorescence microscopy. α-syn, α-synuclein; MPTP, 1-methyl-4-phenyl-1,2,3,6-tetrahydropyridine; M3G, malvidin-3-O-glucoside; ROS, reactive oxygen species. * *p* < 0.05 and **** *p* < 0.0001.

Oxidative stress generated by mitochondrial impairment is a primary factor that contributes to the onset of PD [21]. Increased oxidative stress corresponds to elevated levels of intracellular ROS. Therefore, after confirming the cytoprotective and anti-inflammatory potential of M3G, the antioxidative properties of M3G were further observed. According to the DCFH-DA assay, it was observed that the α-syn individually treated group displayed a non-significant increase in the intracellular ROS levels, which was comparatively enhanced in the presence of MPTP alone. Additionally, higher intracellular ROS levels were observed in the α-syn and MPTP co-treatment group. Surprisingly, the presence of M3G decreased the intracellular ROS levels, indicating the excellent antioxidative ability of M3G against α-syn- and MPTP-induced intracellular ROS levels (Figure 6a). Fluorescence microscopy analysis also displayed a similar pattern, as a higher intensity of fluorescence signals was observed in the α-syn and MPTP co-treatment group, which was again reduced in the presence of M3G (Figure 6b).

### 2.5. M3G Ameliorates Oxidative Stress by Upregulating the Transcriptional Expression Levels of Antioxidants in α-Syn- and MPTP-Induced HMC3 Cells

**Figure 7:**
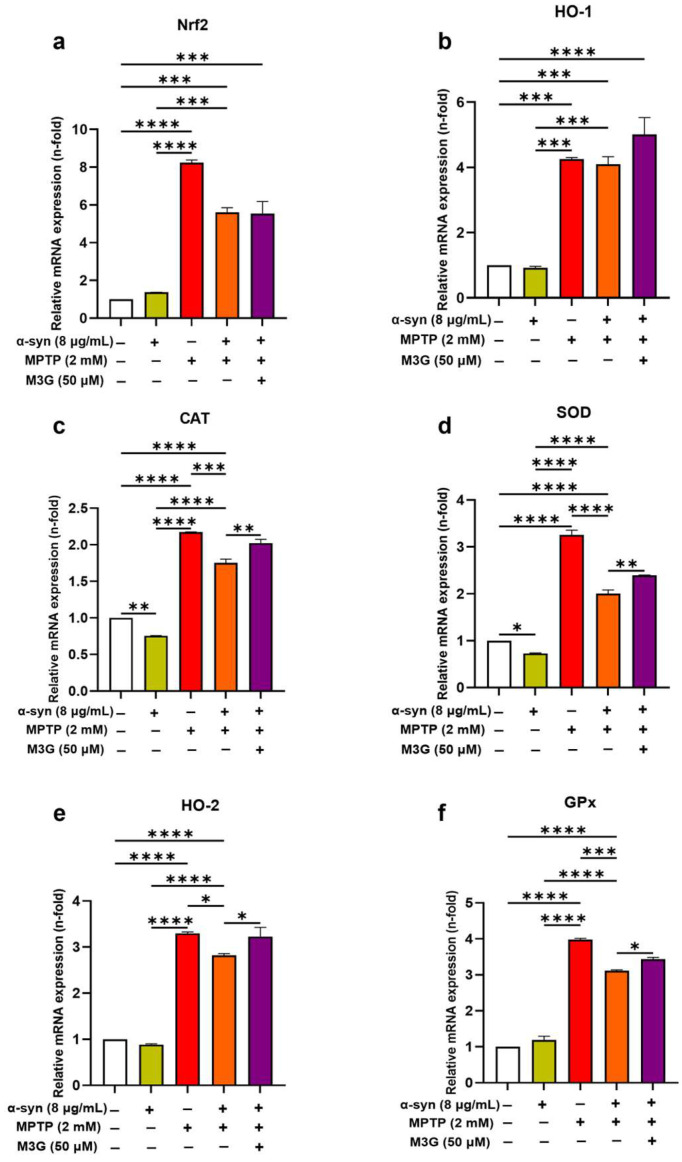
M3G ameliorates the oxidative stress by upregulating the transcriptional expression levels of antioxidants in α-syn- and MPTP-induced HMC3 cells. HMC3 cells were individually or co-treated with α-syn (8 μg/mL) and MPTP (2 mM) in the presence or absence of M3G (50 μM). The relative mRNA expression levels of the antioxidants (**a**) Nrf2, (**b**) HO-1, (**c**) CAT, (**d**) SOD, (**e**) HO-2, and (**f**) GPx were analyzed using RT-PCR. α-syn, α-synuclein; MPTP, 1-methyl-4-phenyl-1,2,3,6-tetrahydropyridine; M3G, malvidin-3-O-glucoside; Nrf2, nuclear factor erythroid 2-related factor 2; HO-1/2, heme oxygenase 1/2; CAT, catalase; SOD, superoxide dismutase; GPx, glutathione peroxidase. Experiments were conducted in triplicate, and all the data are shown as the mean ± standard deviation (SD). * *p* < 0.05, ** *p* < 0.01, *** *p* < 0.001, and **** *p* < 0.0001.

The antioxidative effects of M3G were assessed by examining the mRNA expression levels of various antioxidants, such as Nrf2, HO-1, CAT, SOD, HO-2, and GPx, in the different treatment groups. The mRNA expression levels of Nrf2 and HO-1 did not show any significant change in the M3G treatment group compared to in the co-treatment group; however, the expression levels of Nrf2 and HO-1 were significantly enhanced when compared to the control group (Figure 7a,b). The mRNA expression levels of other antioxidants like CAT, SOD, HO-2, and GPx were found to be significantly upregulated in the presence of M3G compared to in the α-syn and MPTP co-treatment group (Figure 7c–f). These results suggest that the antioxidative action of M3G is effective against α-syn- and MPTP-induced oxidative stress. To determine whether the M3G treatment affects oxidative stress in HMC3 cells, the cells were treated with M3G alone (50 µM). It was determined that the mRNA expression levels of Nrf2, HO-1, HO-2, SOD, CAT, and GPx were upregulated, reflecting its antioxidant properties (Supplementary Figure S2).

### 2.6. M3G Ameliorates Oxidative Stress by Upregulating the Translational Expression Levels of Antioxidants in α-Syn- and MPTP-Induced HMC3 Cells

Nrf2 is a master transcription factor and key regulator of the antioxidant response [36]. During resting metabolic conditions, Nrf2 is present in an inactive form in the cytoplasm, as it binds to Keap1 and is thus prone to proteasomal degradation. However, during oxidative stress, Nrf2 is released from Keap1, resulting in the nuclear translocation of Nrf2. This further leads to the activation of the transcription pathway for transcribing antioxidative and anti-apoptotic genes, which is inhibited in PD pathology [37].

After assessing the antioxidative potential of M3G at the transcriptional level, the effects of M3G were determined by examining the relative protein expression levels of various antioxidants (Nrf2, HO-1, CAT, SOD, HO-2, and GPx) and Keap1 in the α-syn- and MPTP-induced HMC3 cells.

**Figure 8:**
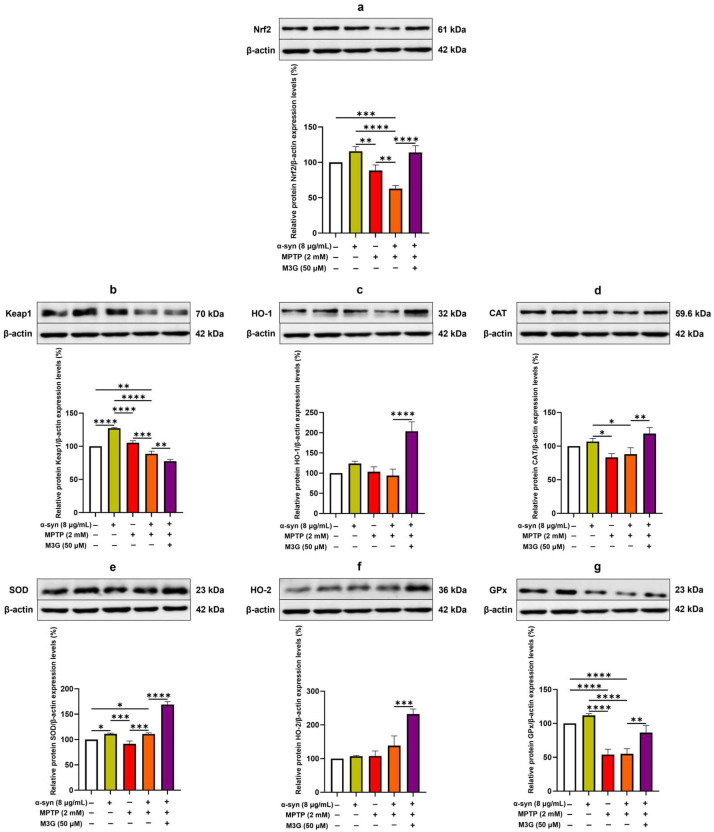
M3G ameliorates oxidative stress by upregulating the translational expression levels of antioxidants in α-syn- and MPTP-induced HMC3 cells. HMC3 cells were individually or co-treated with α-syn (8 μg/mL) and MPTP (2 mM) in the presence or absence of M3G (50 μM). The relative protein expression levels of the antioxidants (**a**) Nrf2, (**b**) Keap1, (**c**) HO-1, (**d**) CAT, (**e**) SOD, (**f**) HO-2, and (**g**) GPx were analyzed using Western blotting. α-syn, α-synuclein; MPTP, 1-methyl-4-phenyl-1,2,3,6-tetrahydropyridine; M3G, malvidin-3-O-glucoside; Nrf2, nuclear factor erythroid 2-related factor 2; Keap1, Kelch-like ECH-associated protein 1; HO-1/2, heme oxygenase 1/2; CAT, catalase; SOD, superoxide dismutase; GPx, glutathione peroxidase. Experiments were conducted in triplicate, and all the data are shown as the mean ± standard deviation (SD). * *p* < 0.05, ** *p* < 0.01, *** *p* < 0.001, and **** *p* < 0.0001.

The protein expression levels of all the antioxidants, such as Nrf2, HO-1, CAT, SOD, HO-2, and GPx (Figure 8a,c–g), were upregulated in the M3G treatment group, when compared to the α-syn and MPTP co-treatment group. The Keap1 protein expression was increased in the α-syn individual treatment group and decreased in the syn and MPTP co-treatment group, which indicated the increased oxidative stress in the α-syn and MPTP co-treatment group, which might have led to the nuclear translocation of Nrf2. However, the Keap1 protein expression was significantly downregulated in the presence of M3G, suggesting the activation of Nrf2/HO-1 signaling (Figure 8b).

Of note, the protein expression levels of Nrf2 (Figure 8a) and HO-1 (Figure 8c) did not follow the same pattern as the mRNA expression level, as a significant increase in their protein expression levels was observed in the M3G treatment group as compared to the α-syn and MPTP co-treatment group. Overall, these results indicated the antioxidative ability of M3G against the α-syn- and MPTP-induced oxidative stress.

### 2.7. M3G Mediates Antioxidative Effects Through Nrf2/HO-1 Signaling

Nrf2/HO-1 signaling is the primary regulator of essential cytoprotective responses and activates the expression of various neuroprotective genes that encode antioxidant, anti-inflammatory, and detoxifying proteins [38]. To determine whether Nrf2/HO-1 signaling is involved in M3G-mediated antioxidative effects, the Nrf2 inhibitor ML385 was used.

**Figure 9:**
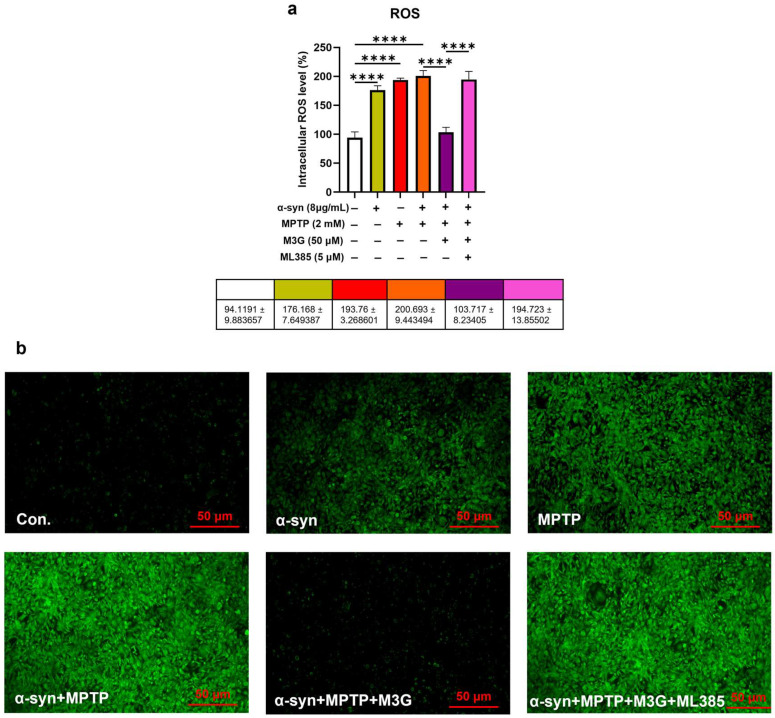
M3G mediates antioxidative effects through Nrf2/HO-1 signaling. HMC3 cells were individually or co-treated with α-syn (8 μg/mL) and MPTP (2 mM) in the presence or absence of M3G (50 μM), with or without ML385 (5 μM). Intracellular ROS levels in α-syn- and MPTP-induced HMC3 cells were determined using the DCFH-DA assay through (**a**) a microplate reader and (**b**) fluorescence microscopy. α-syn, α-synuclein; MPTP, 1-methyl-4-phenyl-1,2,3,6-tetrahydropyridine; M3G, malvidin-3-O-glucoside; ROS, reactive oxygen species. **** *p* < 0.0001.

The results showed that ML385 reversed the antioxidative effects of M3G against the α-syn and MPTP co-treated HMC3 cells, as the oxidative stress (relative intracellular ROS levels) was enhanced in the presence of ML385 (Figure 9a). Fluorescence microscopic analysis was conducted to confirm the results, which also followed a similar pattern as that of ML385, showing increased fluorescence intensity compared to the M3G-treated group (Figure 9b). This inhibitory action of ML385 on the antioxidative potential of M3G suggests that M3G mediates its antioxidative effects via the Nrf2/HO-1 signaling pathway.

### 2.8. M3G Mediates Anti-Apoptotic Effects Through Nrf2/HO-1 Signaling

To further confirm whether mitochondrial ROS are involved in microglial cell death and whether M3G mediates its anti-apoptotic effects via Nrf2/HO-1 signaling, HMC3 cells were co-treated with α-syn (8 μg/mL) and MPTP (2 mM) in the presence or absence of M3G (50 μM), with or without ML385 (5 μM) for 24 h. The relative cell counts of viable and apoptotic cells were observed and calculated by flow cytometry using an annexin V apoptosis assay kit.

**Figure 10:**
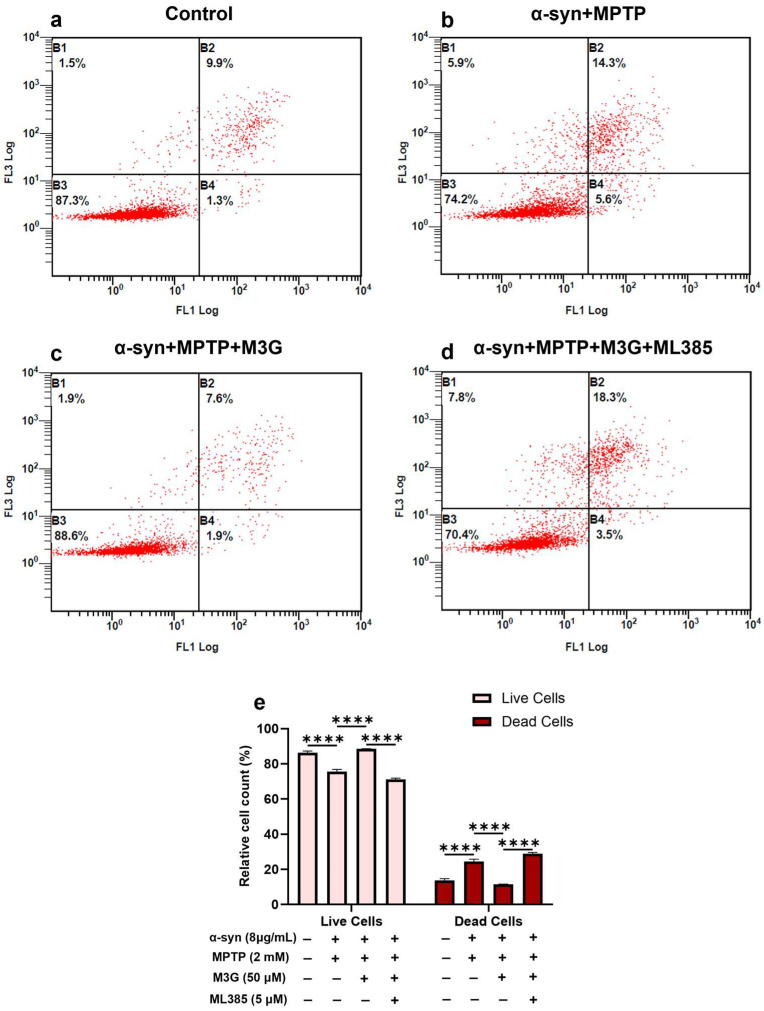
M3G mediates anti-apoptotic effects through Nrf2/HO-1 signaling. (**a**–**e**) HMC3 cells were co-treated with α-syn (8 μg/mL) and MPTP (2 mM) in presence or absence of M3G (50 μM), with/without ML385 (5 μM), and were analyzed for the relative cell count of apoptotic cells using the annexin V–FITC apoptosis assay kit. α-syn, α-synuclein; MPTP, 1-methyl-4-phenyl-1,2,3,6-tetrahydropyridine; M3G, malvidin-3-O-glucoside. Experiments were conducted in triplicate, and all the data are shown as the mean ± standard deviation (SD). **** *p* < 0.0001.

The results revealed that the presence of the Nrf2 inhibitor ML385 reversed the M3G-mediated anti-apoptotic effects, as the relative cell count of live cells was significantly reduced and that of dead cells was significantly increased in the presence of ML385, thus diminishing the M3G’s cytoprotective properties (Figure 10a–e). These results indicate that it might be possible for α-syn and MPTP to induce cell death in HMC3 cells by inducing intracellular ROS, and M3G prevents cells death by activating the Nrf2/HO-1 signaling pathway, thereby reducing ROS through the activation of antioxidants such as HO-1, CAT, SOD, HO-2, and GPx.

## 3. Discussion

Neuronal dysfunction and neuronal cell death are fundamental to neurodegenerative disorders (NDs) [39], and one such disease is PD, which is one of the major contributors to NDs. According to a study on the global burden of disease, PD is potentially the fastest growing neurological disorder worldwide (per the age-standardized rates of disability-adjusted life-years, prevalence, and mortality), thus forming the basis for the Parkinson’s pandemic [40]. Although the exact pathogenesis of PD remains unknown, it has been reported that PD is related to the aggregation of misfolded proteins into Lewy bodies and Lewy neurites in the SNpc region of the midbrain, which are responsible for the death of dopaminergic neurons [41]. In addition to this, the rapid growth in PD incidence and prevalence could be a result of ageing populations [42], decreasing smoking rates (since PD risk is reduced by 40% among smokers) [43], accelerating longevity [44], and industrialization by-products or chemicals, such as trichloroethylene, heavy metals, pesticides (paraquat), solvents, etc. [45]. Hence, the rise in PD incidence could be directly related to the increase in societal growth in terms of gross national income [46]. Considering all these factors, PD is rising as a major health concern globally since it is a strenuous task to examine the trends in the incidence and prevalence of PD because of the constant changes in case definitions over time [47]. The probable risk of PD during the entire lifespan has reached up to one in every fifteen individuals, including for the readers of this paper as well [48].

Dietary anthocyanins have been proactively discussed by various researchers in recent years because of their well-established neuroprotective effects on the central nervous system [49]. These anthocyanins can cross the blood–brain barrier after being absorbed in the form of glycosides in humans and rodents; thereafter, they are confined to different regions of the brain, such as the hippocampus, cortex, striatum, and cerebellum [50]. Strathearn et al. [51] illustrated that anthocyanin- and proanthocyanidin-rich extracts of Chinese mulberry, hibiscus, blueberries, blackcurrant, and grape seeds significantly alleviated rotenone-induced neurodegeneration in PD by enhancing mitochondrial function and interfering with microglial activation. M3G has already been reported for its excellent bioactivities, including the amelioration of oxidative stress, the inhibition of lipid peroxidation in mouse brain homogenates [52], the inhibition of tumor cell growth in the human cell line A-431, and vascular endothelial growth factor (VEGF)-induced angiogenesis [53] in RGC-5 cells by hindering the cAMP pathway, epidermal growth factor receptor (EGFR), and mitogen-activated protein kinase (MAPK) signaling pathway, which is required for normal cell growth and differentiation [54]. It has also been reported to inhibit angiotensin I-converting enzyme (ACE), which results in antihypertensive activity [55], induces apoptosis, and arrests the G2/M phase of the cell cycle in HT-29 colon cancer and human monocytic leukemia cells (U937) [56].

Since PD pathophysiology is difficult to replicate, various mitochondrial neurotoxins, such as MPTP, 6-OHDA, rotenone, and paraquat, are being used for this purpose owing to their extreme neurotoxic properties [57]. Qian et al. [58] reported that anthocyanin-rich blueberry extracts significantly attenuate MPTP-induced behavioral impairment and oxidative stress in PD mice through antioxidative mechanisms. Previously, various MPTP in vivo mouse models were used to mimic PD-related pathophysiology; therefore, this study aimed to develop an in vitro humanized system that correlates the combined effects of α-syn and MPTP and examines the neuroprotective effects of M3G on α-syn- and MPTP-induced cytotoxicity, apoptosis, oxidative stress, and inflammation.

Recent studies have reported that MPTP induces α-syn aggregation in the SNpc region of baboons [9]. Moreover, α-syn knockout mice are resistant to the MPTP-induced degeneration of dopaminergic neurons and dopamine release [59]. Considering these factors, the α-syn and MPTP co-treatment groups were examined extensively in this study. Initially, the cytotoxic effects of α-syn and MPTP on HMC3 cell viability were examined, and it was determined that the α-syn and MPTP co-treatment groups were more toxic to the cells than their individual treatment groups. The optimum cytotoxic concentrations of α-syn and MPTP were 8 μg/mL and 2 mM, respectively, and they were used for further experiments. These results suggest that MPTP increased the α-syn-induced cytotoxic effects but only at 8 μg/mL of α-syn, which could be attributed to MPTP-induced α-syn aggregation, thereby increasing its cytotoxic effect at higher concentrations. Thereafter, it was observed that the cytoprotective effect of M3G was dose-dependent, with a 50 µM concentration of M3G being an ideal concentration, owing to its appreciable effect on maintaining normal cell viability in the presence of α-syn and MPTP. This is consistent with the previously published literature in which 50 µM of malvidin was found to be cytoprotective against LPS-induced RAW 264.7 macrophages [28].

In PD, microglia-mediated apoptosis plays a vital role in the gradual and progressive loss of dopaminergic neurons in the SNpc, resulting in the hallmark motor symptoms. The pro-apoptotic markers casp-3 and casp-8 are responsible for the different apoptotic pathways implicated in PD. The executioner caspase (casp-3) cleaves different cellular components, leading to apoptosis [60]. The initiator caspase (casp-8) provokes an extrinsic apoptotic pathway, leading to cell death in PD [61]. Additionally, the pro-apoptotic protein Bax promotes apoptosis and mitochondrial dysfunction by assisting cytochrome c release, whereas the anti-apoptotic protein Bcl-2 inhibits this effect by inhibiting Bax activity [62]. In PD, an imbalance between Bax and Bcl-2 is critical in determining the vulnerability of dopaminergic neurons to apoptosis. In this study, the M3G treatment significantly downregulated the transcriptional expression levels of the pro-apoptotic markers Bax, casp-3, and casp-8 and significantly upregulated the expression of the anti-apoptotic marker Bcl-2. M3G also reduced the protein expression of cleaved casp-3/pro-casp-3 and Bax while inducing Bcl-2 expression. These results are similar to those of previously published studies on the anti-apoptotic effects of M3G [63]. Additionally, the concurrent activation of both caspases (casp-3 and casp-8) suggests that both intrinsic (mitochondrial) and extrinsic (death-receptor-mediated) pathways may be involved in the apoptosis of HMC3 cells upon co-treatment with α-syn and MPTP. Understanding this dual activation is crucial, as it highlights the complexity of the apoptotic response in neuroinflammatory conditions and suggests that targeting both pathways may be necessary for effective neuroprotection.

**Scheme 1:**
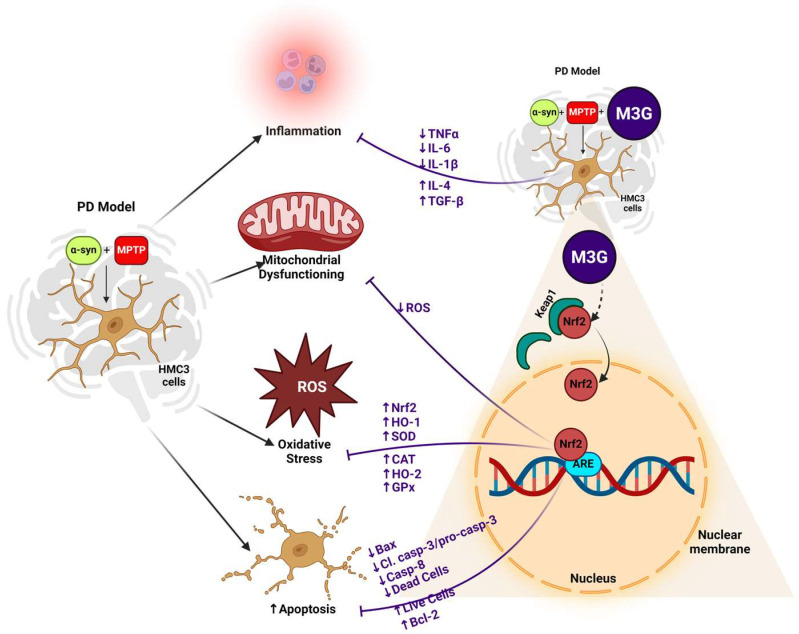
Malvidin-3-O-glucoside ameliorates α-syn and MPTP co-induced cytotoxicity in HMC3 cell model of Parkinson’s disease. PD, Parkinson’s disease; HMC3, human microglial cells; α-syn, α-synuclein; MPTP, 1-methyl-4-phenyl-1,2,3,6-tetrahydropyridine; M3G, malvidin-3-O-glucoside; IL, interleukin; TNF-α, tumor necrosis factor-alpha; TGF-β, transforming growth factor-beta; ROS, reactive oxygen species; SOD, superoxide dismutase; CAT, catalase; Nrf2, nuclear factor erythroid 2-related factor 2; HO-1/2, heme oxygenase 1/2; GPx, glutathione peroxidase; Keap1, Kelch-like ECH-associated protein 1; ARE, antioxidant responsive element; Bax, Bcl-2-Associated X-protein; Bcl2, B-cell lymphoma 2; Casp, caspase; ↑, upregulation; ↓, downregulation.

Both neuroinflammation and innate immunity have been considered to play important roles in the onset and progression [64]. A large body of evidence suggests that pro-inflammatory cytokines might play a role in mediating neuronal degeneration [65]. In this study, the mRNA expression levels of various pro-inflammatory cytokines such as IL-1β, TNF-α, and IL-6 were assessed by treating HMC3 cells with α-syn and MPTP either individually or in combination with or without M3G. M3G was able to significantly ameliorate the α-syn- and MPTP-induced release of these pro-inflammatory cytokines and significantly upregulate the expression of anti-inflammatory cytokines like IL-4 and TGF-β (Scheme 1), thus supporting the role of M3G as a potential anti-inflammatory agent, which is consistent with previous findings by Huang et al. [66], where M3G attenuated the LPS-induced upregulation of pro-inflammatory cytokines in RAW 264.7 macrophages and downregulated the TNF-α-induced inflammation in the endothelial cells.

Recent studies suggest that dopaminergic neurons might be inherently vulnerable to reactive microgliosis, providing much-needed insights into the chronic progression of PD [67]. Additionally, mitochondrial dysfunction accelerates dopaminergic neuronal cell death in the SNpc along with oxidative stress, resulting in reduced voluntary movement in PD, which might play a key role in neurodegeneration [68]. Oxidative stress results from ROS generation during oxidative phosphorylation or dopamine metabolism [69]. A study of a PD brain reported that GPx levels are reduced in patients with PD. Moreover, oxidative stress and damage have been observed in the DNA, proteins, and lipids [70]. In PD, MPTP decreases mitochondrial complex I activity and enhances ROS production [71]. MPP+, an MPTP metabolite, is assumed to be responsible for mediating the neurotoxic effects of MPTP because it inhibits mitochondrial complex I at or near the binding site of rotenone [72]. Oxidative stress can also play a major role in α-syn-mediated pathology in PD, as normal α-syn protein is less susceptible to aggregation than the oxidatively modified α-syn protein [73]. In addition to this, the increased expression of α-syn can itself contribute to oxidative stress [74].

Considering all these factors, the mRNA and protein expression levels of various antioxidants in α-syn and MPTP co-treated HMC3 cells in the presence of M3G were assessed. The mRNA and protein expression levels of all the antioxidants such as CAT, SOD, HO-2, and GPx were increased, but in the case of Nrf2 and HO-1, only the protein expressions were increased in the M3G-treated group compared to in the α-syn and MPTP co-treatment group. This could be because mRNA and protein can be expressed at different time intervals. These results are consistent with the previously reported antioxidative properties of M3G in endothelial cells and non-alcoholic fatty liver disease [75]. The cysteine residues of Keap1 are modified in the presence of oxidative stress (ROS), electrophiles, and reactive nitrogen species (RNS), leading to the inactivation of Keap1 and the subsequent stabilization of Nrf2. Nrf2 then translocates to the nucleus, resulting in the increased expression of antioxidative response element genes. Consistent with previous findings [37], in this study, the Keap1 protein expression levels were found to be elevated in the α-syn-only group, which was reduced in the presence of M3G, thus confirming the nuclear translocation of Nrf2. Surprisingly, the Keap1 expression in the α-syn and MPTP co-treatment group was reduced significantly when compared to the α-syn-only group. This could be attributed to the overly enhanced oxidative stress in the α-syn and MPTP co-treatment group, which might have led to the inactivation of Keap1 and the ultimate nuclear translocation of Nrf2. Additionally, when the ROS levels were assessed in the different treatment groups, it was found that M3G was able to significantly ameliorate the α-syn- and MPTP-induced oxidative stress in the HMC3 cells, thus again confirming M3G’s antioxidative properties (Scheme 1). These results are similar to the previously published literature displaying M3G’s ability to ameliorate mitochondrial dysfunction and ROS accumulation as a result of LPS stimulation [76].

The Nrf2 inhibitor ML385 has been reported to inhibit Nrf2 DNA binding, relative expression, and downstream target genes [77]. When the Nrf2 inhibitor ML385 was co-treated in the α-syn, MPTP, and M3G co-treatment group, the oxidative stress was found to be significantly upregulated, which was reduced in the treatment group without ML385. Similarly, ML385 enhanced the relative cell count of apoptotic cells, which was reduced by the presence of M3G in α-syn and MPTP co-treated HMC3 cells. These results confirmed that the cytoprotective (antioxidative and anti-apoptotic) properties of M3G in the α-syn and MPTP co-treated HMC3 cells are mediated through Nrf2/HO-1 signaling, which is consistent with the previously reported literature regarding the Nrf2/HO-1-mediated antioxidative properties of M3G and the inhibitory action of ML385 [78].

To the best of our knowledge, this is the first study to design a humanized in vitro PD-like pathology using α-syn and MPTP and illustrate the effects of M3G in ameliorating α-syn- and MPTP-induced cytotoxicity, apoptosis, and oxidative stress, in an in vitro humanized PD model. However, both in vitro and in vivo studies should be conducted to elucidate the mechanism underlying the M3G-mediated neuroprotective properties in a PD model. Although it is known that α-syn oligomer formation [79], proteasomal and mitochondrial dysfunction [80], increased membrane conductance [81], and microglial activation [79] are the major reasons for α-syn-induced cell death in various cell culture models, all these factors need to be thoroughly assessed in an α-syn and MPTP co-treated in vitro and in vivo PD model in the presence of M3G. In addition, the accumulation of intracellular proteins due to the failure of the ubiquitin–proteasome system (UPS) is common in both familial and sporadic PD and could cause or contribute to the initiation and/or progression of nigrostriatal degeneration in these disorders [82]. Therefore, the UPS activity, including the levels of ubiquitinated proteins and the expression of UPS components following α-syn and MPTP co-treatment in the presence or absence of M3G, should be analyzed in future studies and can be seen as the limitation of this study. Recent studies have shown that in addition to α-syn fibrillation, there are other molecular mechanisms mediated by different peptides, such as Aβ peptide, and tau protein that might come into play at the later stages of PD. The cross-seeding of these peptides could be a novel molecular mechanism underlying the later stages of PD pathology. The Aβ peptide, a key pathological hallmark of AD, promotes the seeding and spreading of α-syn and tau in a mouse model of Lewy body disorders with Aβ pathology [83] and has also been reported to induce α-syn oligomerization [84]. Likewise, the Lewy bodies (pathological α-syn aggregates) have been reported to be a common co-pathology in AD, along with Aβ plaque and neurofibrillary tau [85]. These factors need to be explored thoroughly in future studies to identify the inter-relationships among different neurodegenerative disorders and to ensure that a novel compound effective against all these inter-related pathologies can be identified.

## 4. Materials and Methods

### 4.1. Materials

The HMC3 (ATCC^®^ CRL-3304™) cell line used in this study was obtained from American Type Culture Collection (ATCC; Manassas, VA, USA). The human reactive rabbit monoclonal recombinant anti-Kelch-like ECH-associated protein 1 (Keap1, ab227828) antibody and recombinant human alpha-synuclein protein (ab51189) were provided by Abcam (Cambridge, UK). M3G (CFN92049) was provided by ChemFaces (Wuhan, China). MPTP hydrochloride (HY-15608) and Nrf2 inhibitor ML385 (HY-100523) were obtained from MedChemExpress LLC (Princeton, NJ, USA). Dulbecco’s modified Eagle medium (DMEM, basal medium), antibiotic–antimycotic (penicillin/streptomycin) solution, phosphate-buffered saline (PBS, isotonic solution), fetal bovine serum (FBS), and trypsin were obtained from Thermo Fisher Scientific (Waltham, CA, USA). Santa Cruz Biotechnology (Dallas, TX, USA) provided the humanized mouse monoclonal primary antibodies for nuclear factor erythroid 2-related factor 2 (Nrf2, sc-365949, A-10), heme oxygenase 1 (HO-1, sc-136960, A-3), heme oxygenase 2 (HO-2, sc-17786, B-3), superoxide dismutase 1 (SOD1, sc-101523, 24), glutathione peroxidase 1/2 (GPX1/2, sc-133160, B-6), and β-actin (sc-47778, 1:5000). Catalase (CAT; CF502564) mouse monoclonal antibody was purchased from OriGene (Rockville, MD, USA). Human reactive primary antibodies against Bcl-2 (2876S), Bax (2772S), caspase-3 (9662S), cleaved caspase-3 (9661S), and caspase-8 (9746S) were obtained from Cell Signaling Technology (Danvers, MA, USA).

### 4.2. HMC3 Cell Culture

The immortalized human microglial cell line HMC3 was cultured in DMEM supplemented with 1% antibiotic–antimycotic (penicillin/streptomycin) solution and 10% heat-inactivated FBS and maintained at 37 °C in a 5% CO_2_ incubator. M3G was prepared in DMSO; therefore, DMSO was used as a control in all experimental treatment groups with M3G.

### 4.3. Cell Viability and Cytotoxicity Assay Protocol

A Cell Counting Kit-8 assay (Dojindo Molecular Technologies, Inc., Rockville, MD, USA) was used to measure HMC3 cell viability in accordance with the manufacturer’s guidelines. Briefly, HMC3 cells were seeded at a density of 1 × 10^4^ cells/well in a 96-well cell culture plate, allowed to adhere, and grown in a 5% CO_2_ incubator at 37 °C for 24 h. Three treatment groups were then established: the individual treatment group where the cells were treated with various concentrations of α-syn (0.5, 1, 2, 4, and 8 μg/mL), MPTP (0.25, 0.5, 1, and 2 mM), and M3G (6.25, 12.5, 25, 50, and 100 μM) individually; the co-treatment group in which the cells were treated with MPTP (2 mM) in the presence of different concentrations of α-syn (0.5, 1, 2, 4, and 8 μg/mL); and the M3G co-treatment group with MPTP (2 mM), α-syn (8 μg/mL), and different concentrations of M3G (6.25, 12.5, 25, 50, and 100 μM). All treatments were conducted for 24 h. The culture medium was aspirated, and the cells were treated with the described amount of CCK-8 solution and incubated for 2 h. The optical density of the samples was measured at 450 nm using a microplate spectrophotometer (BioTek Inc., Winooski, VT, USA). The percentage of viable cells was calculated to quantify the total growth inhibition, with the control cells presumed to be 100% viable.

### 4.4. RNA Preparation and Real-Time PCR

HMC3 cells were seeded at a density of 1 × 10^6^ cells/well in a 6-well cell culture plate and allowed to adhere and grow at 37 °C for 24 h. The cells were then treated with α-syn (8 μg/mL) and MPTP (2 mM) individually or in a co-treatment with α-syn (8 μg/mL) and MPTP (2 mM), in the presence or absence of M3G (50 μM). An RNA extraction kit (iNtRON Biotechnology, Gyeonggi-do, Republic of Korea) was used to extract total RNA according to the manufacturer’s guidelines. Reverse transcription of 50 ng RNA to complementary DNA was performed using PCR (TaKaRa Bio, Kusatsu, Shiga, Japan). RT-PCR was performed with TB Green (TaKaRa Bio, Kusatsu, Shiga, Japan) using an ABI QuantStudio3 PCR system (Applied Biosystems, Foster City, CA, USA). All experiments were performed in triplicate. The specific primer sets used for PCR amplification of the target genes in HMC3 cells are listed in Table 1. β-actin gene was used as an endogenous control for normalizing gene expression.

### 4.5. 2′,7′-Dichlorodihydrofluorescein Diacetate (DCFH-DA/H2DCFDA) Assay

DCFH-DA is a membrane-permeable compound that can be enzymatically converted to 2′,7′-dichlorofluorescein (DCF), a highly fluorescent compound, in the presence of ROS. To examine the antioxidant properties of M3G, HMC3 cells were seeded at a density of 2.5 × 10^4^ cells/well in a black 96-well clear-bottom plate and incubated at 37 °C for 24 h. The cell culture medium was then aspirated, followed by individual treatment with α-syn (8 μg/mL) and MPTP (2 mM) and co-treatment with α-syn (8 μg/mL) and MPTP (2 mM) in the presence or absence of M3G (50 μM) for 24 h. The culture medium was then aspirated again, and the cells were treated with 2′,7′-DCFH-DA for 45 min. This experiment was repeated in the presence of an Nrf2 inhibitor (ML385; 5 μM). The HMC3 cells were treated with α-syn (8 μg/mL) and MPTP (2 mM) individually and co-treated with α-syn (8 μg/mL) and MPTP (2 mM) in the presence or absence of M3G (50 μM), with/without ML385. A microplate reader was used to monitor the fluorescence at excitation and emission wavelengths of 488 and 525 nm, respectively. Fluorescence was examined using a fluorescence microscope (Olympus, Tokyo, Japan). The results are expressed as a percentage relative to DCF fluorescence in the respective control cells.

### 4.6. Annexin Valinomycin–Fluorescein Isothiocyanate (V-FITC) Apoptosis Detection Assay

An annexin V–FITC apoptosis staining/detection kit was used for flow cytometry-based detection of apoptotic cells by staining phosphatidylserine molecules that had translocated to the outside of the cell membrane. This kit is used to distinguish between necrosis and apoptosis when performing annexin V–FITC and propidium iodide (PI) staining. After the initiation of apoptosis, the cells quickly translocate phosphatidylserine molecules from the inner portion of the cell membrane to the cell surface. Thereafter, they are stained with a fluorescent conjugate of annexin V (a protein with a high affinity for phosphatidylserine) to detect the presence of phosphatidylserine on the cell surface. The combination of annexin V and PI permits the differentiation of viable cells, which are annexin V-negative and PI-negative; early apoptotic cells are annexin V-positive and PI-negative, and necrotic cells are annexin V-positive and PI-positive. Briefly, HMC3 cells were co-treated with α-syn (8 μg/mL) and MPTP (2 mM) in the presence or absence of M3G (50 μM) for 24 h and harvested thereafter. Similarly, while experimenting with the Nrf2 inhibitor, HMC3 cells were again co-treated with α-syn (8 μg/mL) and MPTP (2 mM) in the presence or absence of M3G (50 μM), with/without the presence of the Nrf2 inhibitor (ML385; 5 μM). The harvested cells were washed with Dulbecco’s phosphate-buffered saline (DPBS), and each group was divided into duplicate groups, including the control. The cells were suspended in 500 µL 1X binding buffer, containing 5 µL annexin V–FITC and 5 µL PI for each sample in the dark for 10 min at room temperature. Viable and apoptotic cells were detected by flow cytometry using an FC500 MLP cytometer (Beckman Coulter Inc., Brea, CA, USA).

### 4.7. Western Blotting

HMC3 cells were seeded at a density of 1 × 10^6^ cells/well in a 6-well cell culture plate for 24 h and then treated with α-syn (8 μg/mL) and MPTP (2 mM) individually or co-treated with α-syn (8 μg/mL) and MPTP (2 mM) in the presence or absence of M3G (50 μM). To analyze protein expression, a protein lysis buffer (iNtRON Biotechnology Gyeonggi-do, Republic of Korea) containing protease and phosphate inhibitors was used to harvest the total protein content from HMC3 cells. Forty micrograms of protein samples was separated using sodium dodecyl sulfate–polyacrylamide gel electrophoresis (SDS-PAGE) on a polyacrylamide gel containing 10% SDS and then electrophoretically shifted to polyvinylidene difluoride (PVDF) membranes (Bio-Rad Laboratories, Hercules, CA, USA) overnight. Thereafter, 5% skim milk was used for blocking at room temperature (RT, 18 to 25 °C) for 1 h, and the membranes were then washed thrice with 1X Tris-Buffered Saline (TBST) and incubated again with the subsequent primary antibodies at RT for 2 h followed by an overnight incubation at 4 °C: anti-Bax (1:1000), anti-Bcl-2 (1:1000), anti-pro-casp-3 (1:500), anti-cleaved casp-3 (1:1000), anti-casp-8 (1:500), anti-Nrf2 (1:500), anti-Keap1 (1:2000), anti-HO-1 (1:1000), anti-CAT (1:500), anti-SOD1 (1:500 dilution), anti-HO-2 (1:1000), anti-GPx (1:500), and anti-β-actin (1:5000). The membranes were washed and incubated with the corresponding secondary antibodies for 1 h at RT. The blots of the target proteins were developed using the Miracle-Star™ Western Blot Detection System (iNtRON Biotechnology, Seongnam-si, Republic of Korea), and the reactive band signals were visualized using the ImageQuantTM LAS500 system (GE Healthcare Life Sciences, Issaquah, WA, USA). In a few cases, the membrane blots were stripped by submersion in stripping buffer (iNtRON Biotechnology, Seongnam, Republic of Korea) and maintained at 37 °C in an incubator covered with aluminum foil. The membrane blots were washed after 25 min of incubation using 1X TBST thrice at 10 min intervals, blocked for 1 h, and re-probed by incubating with another primary antibody for 2 h at room temperature followed by an overnight incubation at 4 °C. The membranes were washed and incubated with the corresponding secondary antibodies for 1 h at room temperature. Amersham Imager 680 analysis software (GE Healthcare Life Sciences, Piscataway, NJ, USA) was used to obtain and analyze the densitometry data after the Western blot analysis.

### 4.8. Statistical Analysis

All experiments were conducted a total of at least three times, and all the data are shown as the mean ± standard deviation (SD). GraphPad Prism 10.1.2 (GraphPad Software Inc., San Diego, CA, USA) was used to analyze significant differences between various treatment groups. One-way ANOVA (analysis of variance) was used to compare the variations among the groups, and Tukey’s post hoc test was used to assess and compare the results. Variance was considered to be of statistical significance with a *p* value of <0.05.

## 5. Conclusions

In conclusion, a humanized in vitro PD-like pathology model using α-syn and MPTP was developed. Furthermore, the potential utility of anthocyanin M3G as a therapeutic agent in the treatment of PD was established. In this study, M3G was reported to exert anti-apoptotic, anti-inflammatory, and antioxidative properties against the α-syn- and MPTP-induced cytotoxicity in HMC3 cells. The effective cellular defense mechanism against α-syn- and MPTP-induced apoptosis was observed via the downregulated expression levels of the pro-apoptotic markers Bax, cleaved casp-3/pro-casp-3, and casp-8 and the upregulated expression levels of the anti-apoptotic marker Bcl-2 in the M3G-treated group. Likewise, antioxidative effects were observed via the upregulated transcriptional and translational expression of the antioxidants Nrf2, CAT, SOD, HO-1/2, and GPx in the presence of M3G. M3G reduced the anti-apoptotic and antioxidant properties in the presence of the Nrf2 inhibitor ML385, revealing the role of Nrf2/HO-1 signaling in the antioxidative and anti-apoptotic actions of M3G. These results indicate that M3G has antioxidative and anti-apoptotic properties and contributes to eliminating the α-syn- and MPTP-induced inflammatory stress. However, further in vitro and in vivo studies are required to fully understand the mechanism of action of M3G-mediated cytoprotection.

## Figures and Tables

**Table 1:** List of forward and reverse primer sets used for PCR amplification of the target genes in HMC3 cells.

Target Gene	Forward Primer Sequence	Reverse Primer Sequence
β-actin	5′-CTCTTCCAGCCTTCCTTCCT-3′	5′-AGCACTGTGTTGGCGTACAG-3′
Nrf2	5′-TCCGGGTGTGTTTGTTCCAA-3′	5′-CGCCCGCGAGATAAAGAGTT-3′
HO-1	5′-AGACTGCGTTCCTGCTCAAC-3′	5′-AAAGCCCTACAGCAACTGTCG-3′
CAT	5′-GTGCGGAGATTCAACACTGCCA-3′	5′-CGGCAATGTTCTCACACAGACG-3′
SOD	5′-CTCACTCTCAGGAGACCATTGC-3′	5′-CCACAAGCCAAACGACTTCCAG-3′
HO-2	5′-CCACCACGGCACTTTACTTCA-3′	5′-CGTTCTGCCCTATGTAGTGGA-3′
GPx	5′-GTGCTCGGCTTCCCGTGCAAC-3′	5′-CTCGAAGAGCATGAAGTTGGGC-3′
IL-1β	5′-GGGATAACGAGGCTTATGTGC-3′	5′-AGGTGGAGAGCTTTCAGTTCA-3′
IL-6	5′-GACCCAACCACAAATGCCAG-3′	5′-GAGTTGTCATGTCCTGCAGC-3′
TNF-α	5′-TGAGCACTGAAAGCATGATCC-3′	5′-GGAGAAGAGGCTGAGGAACA-3′
IL-4	5′-CCGTAACAGACATCTTTGCTGCC-3′	5′-GAGTGTCCTTCTCATGGTGGCT-3′
TGF-β	5′-TACCTGAACCCGTGTTGCTCTC-3′	5′-GTTGCTGAGGTATCGCCAGGAA-3′
Bax	5′-TCAGGATGCGTCCACCAAGAAG-3′	5′-TGTGTCCACGGCGGCAATCATC-3′
Bcl2	5′-ATCGCCCTGTGGATGACTGAGT-3′	5′-GCCAGGAGAAATCAAACAGAGGC-3′
Casp-3	5′-GGAAGCGAATCAATGGACTCTGG-3′	5′-GCATCGACATCTGTACCAGACC-3′
Casp-8	5′-AGAAGAGGGTCATCCTGGGAGA-3′	5′-TCAGGACTTCCTTCAAGGCTGC-3′

TNF-α, tumor necrosis factor-alpha; IL, interleukin; Nrf2, nuclear factor erythroid 2-related factor 2; HO-1/2, heme oxygenase 1/2; CAT, catalase; SOD, superoxide dismutase; GPx, glutathione peroxidase; TGF-β, transforming growth factor-beta; Bax, Bcl-2-Associated X-protein; Bcl2, B-cell lymphoma 2; Casp, caspase.

## Data Availability

All data and results obtained in the current study are available from the corresponding author upon reasonable request.

## Supplementary Materials

The following supporting information can be downloaded at https://www.mdpi.com/article/10.3390/ijms252312733/s1.
