# Supplementary material for: Malvidin-3-O-Glucoside Mitigates α-Syn and MPTP Co-Induced Oxidative Stress and Apoptosis in Human Microglial HMC3 Cells
Source: Int J Mol Sci. 2024 Nov 27;25(23):12733. doi: 10.3390/ijms252312733 (PMC11641650; doi:10.3390/ijms252312733)
Supplement: Supplementary file 1 [file ijms-25-12733-s001.zip › ijms-3228754-supplementary.pdf]

## Supplementary Materials

# Malvidin-3-O-glucoside mitigates $\alpha$-syn and MPTP co-induced oxidative stress and apoptosis in human microglial HMC3 cells

Rachit Sood[1,2], Sanjay [1,2], Sung-Ung Kang[3,4], Na Young Yoon[5] and Hae-Jeung Lee[1,2,6*]

1. Department of Food and Nutrition, College of BioNano Technology, Gachon University, Seongnam, Gyeonggi-do 13120, Republic of Korea (R.S., rachitsood1998@gmail.com)
2. Institute for Aging and Clinical Nutrition Research, Gachon University, Seongnam, Gyeonggi-do 13120, Republic of Korea (S., sanjay.monga4@gmail.com)
3. Neuroregeneration and Stem Cell Programs, Institute for Cell Engineering, Johns Hopkins University School of Medicine, Baltimore, MD 21205, USA
4. Department of Neurology, Johns Hopkins University School of Medicine, Baltimore, MD 21205, USA (S-U.K., skang34@jhmi.edu)
5. Food Safety and Processing Research Division, National Institute of Fisheries Science, Busan 46083, Republic of Korea (N.Y.Y., dbssud@korea.kr)
6. Department of Health Sciences and Technology, Gachon Advanced Institute for Health Science and Technology (GAIHST), Gachon University, Incheon 21999, Republic of Korea (H. -J. L., skysea@gachon.ac.kr)

***Correspondence:** skysea@gachon.ac.kr or skysea1010@gmail.com (H.-J.L.)

Tel.: +82-31-750-5968 (H.-J.L.); Fax: +82-31-724-4411 (H.J.-L.)

*** Correspondence**

Prof. Hae-Jeung Lee
College of Bionano Technology, Dept. of Food & Nutrition, Gachon University.
1342 Seongnamdaero, Sujeong-gu, Seongnam-si, Gyeonggi-do, South Korea.
Postal code: 13120.
Tel: +82-31-750-5968; Fax: +82-31-750- 5974
Email: skysea@gachon.ac.kr, skysea1010@gmail.com

**Supplementary Fig. S1**

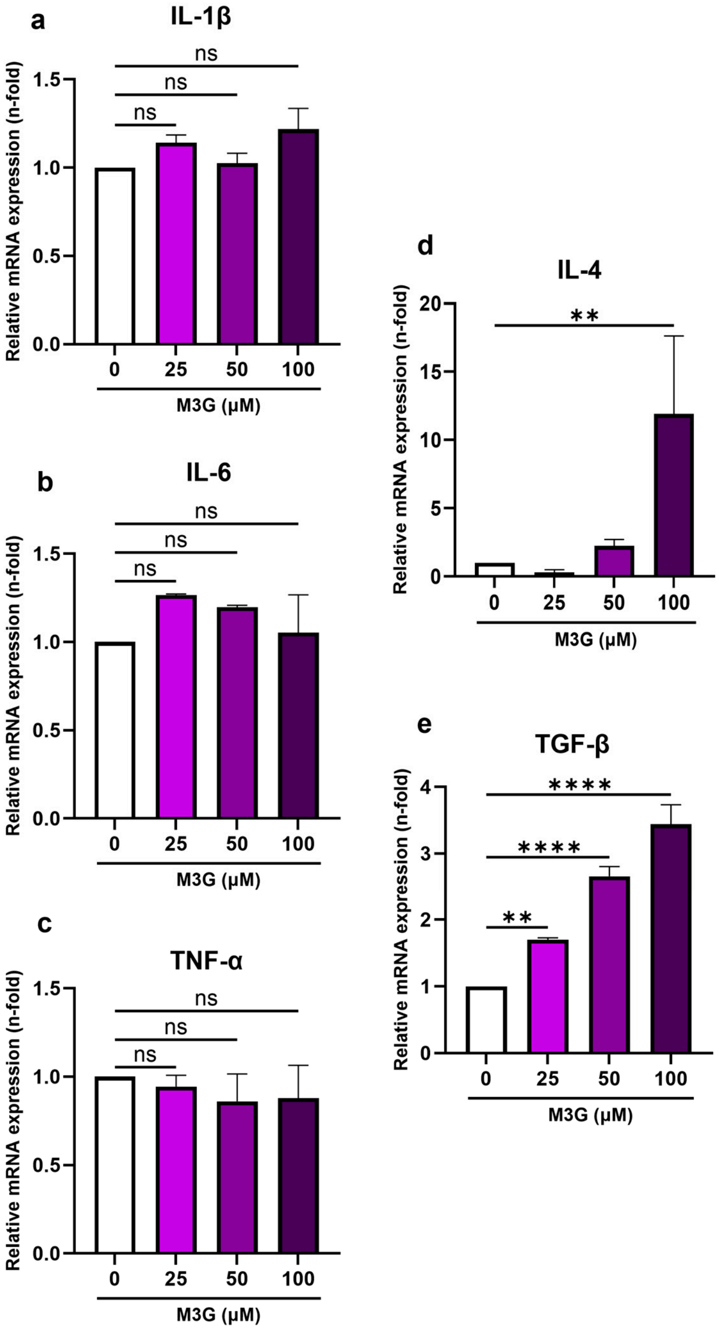


**Figure S1. M3G alone displays anti-inflammatory properties.** HMC3 cells were treated with M3G (50 μM) alone for 24 h, and the mRNA expression of the pro-inflammatory cytokines: (a) IL-1β, (b) IL-6 and (c) TNF-$\alpha$, and the anti-inflammatory cytokines: (d) IL-4 and (e) TGF-β was analyzed by RT-PCR. M3G, malvidin-3-O-glucoside; IL, interleukin; TNF-$\alpha$, tumor necrosis factor-alpha; TGF-β, transforming growth factor-beta. The experiments were conducted for a total of at least three times, and all the data is shown as the mean ± standard deviation (SD). $^{**}p < 0.01$ and $^{****}p < 0.0001$.

**Supplementary Fig. S2**

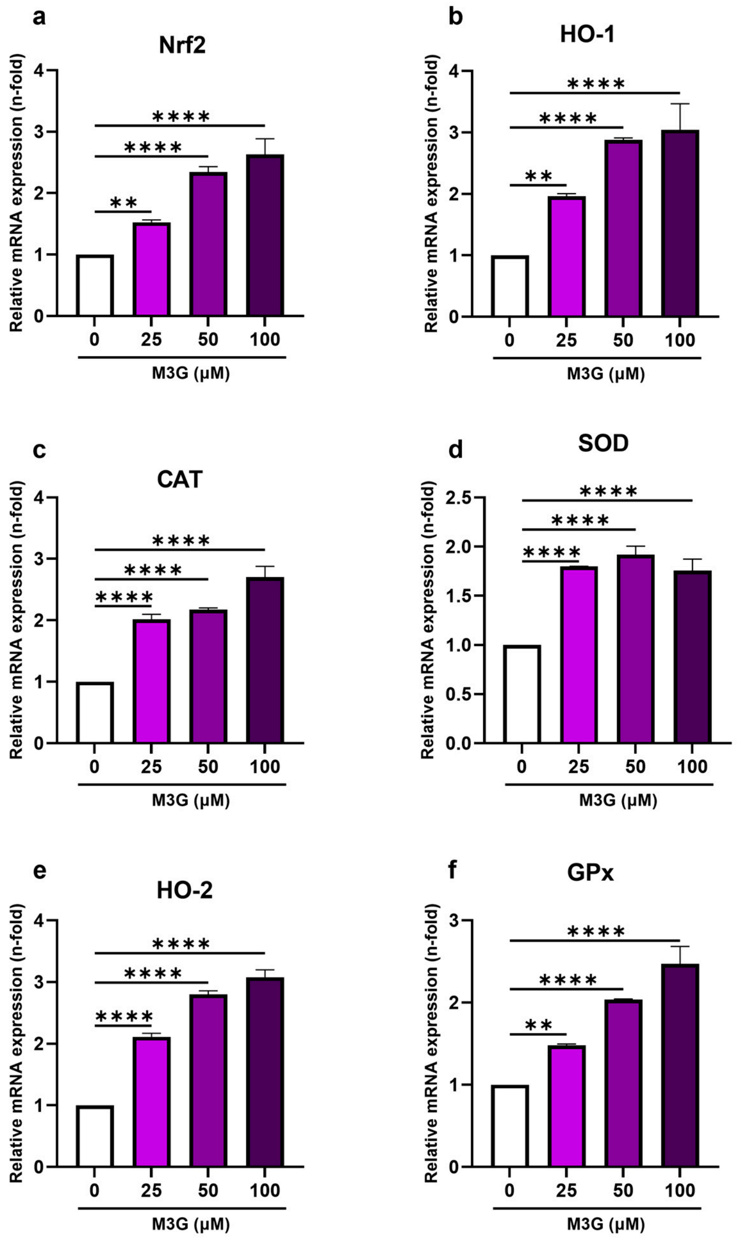


**Figure S2. M3G alone displays anti-oxidative properties.** HMC3 cells were treated with M3G (50 μM) alone for 24 h, and the relative mRNA expressions levels of the antioxidants: (a) Nrf2, (b) HO-1, (c) CAT, (d) SOD, (e) HO-2, and (f) GPx were analyzed using RT-PCR. M3G, malvidin-3-O-glucoside; Nrf2, nuclear factor erythroid 2-related factor 2; HO-1/2, heme oxygenase-1/2; CAT, catalase; SOD, superoxide dismutase; GPx, glutathione peroxidase. The experiments were conducted for a total of at least three times, and all the data is shown as the mean ± standard deviation (SD). $^{**}p < 0.01$ and $^{****}p < 0.0001$.
